# Enhanced hydrogenation catalyst synthesized by *Desulfovibrio desulfuricans* exposed to a radio frequency magnetic field

**DOI:** 10.1111/1751-7915.13878

**Published:** 2021-07-03

**Authors:** Lynne E. Macaskie, John Collins, Iryna P. Mikheenko, Jaime Gomez‐Bolivar, Mohamed L. Merroun, James A. Bennett

**Affiliations:** ^1^ School of Biosciences University of Birmingham Edgbaston, Birmingham B15 2TT UK; ^2^ C‐Tech Innovation Ltd. Capenhurst Technology Park Capenhurst CH1 6EH UK; ^3^ Department of Microbiology Faculty of Sciences University of Granada Campus Fuentenueva Granada 18071 Spain

## Abstract

*Desulfovibrio desulfuricans* reduces Pd(II) to Pd(0)‐nanoparticles (Pd‐NPs) which are catalytically active in 2‐pentyne hydrogenation. To make Pd‐NPs, resting cells are challenged with Pd(II) ions (uptake), followed by addition of electron donor to promote bioreduction of cell‐bound Pd(II) to Pd(0) (bio‐Pd). Application of radiofrequency (RF) radiation to prepared 5 wt% bio‐Pd catalyst (60 W power, 60 min) increased the hydrogenation rate by 70% with no adverse impact on selectivity to *cis‐*2‐pentene. Such treatment of a 5 wt% Pd/carbon commercial catalyst did not affect the conversion rate but reduced the selectivity. Lower‐dose RF radiation (2–8 W power, 20 min) was applied to the bacteria at various stages before and during synthesis of the bio‐scaffolded Pd‐NPs. The reaction rate (μ mol 2‐pentyne converted s^‐1^) was increased by ~threefold by treatment during bacterial catalyst synthesis. Application of RF radiation (2 or 4 W power) to resting cells prior to Pd(II) exposure affected the catalyst made subsequently, increasing the reaction rate by 50% as compared to untreated cells, while nearly doubling selectivity for *cis* 2‐pentene. The results are discussed with respect to published and related work which shows altered dispersion of the Pd‐NPs made following or during RF exposure.

## Graphical abstract

*Desulfovibrio desulfuricans* makes catalytically active palladium nanoparticles (bio‐Pd). The catalytic efficacy in hydrogenation of 2‐pentyne is enhanced by radiofrequency (RF) irradiation of the bio‐Pd once synthesised, or by pre‐injury of the bacteria via an RF field that predisposes them to make a better catalyst subsequently.

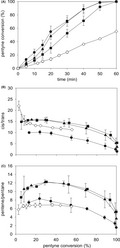

## Introduction

Bacteria can reduce precious metals, forming metallic nanoparticles (NPs) in cell surface layers (Mikheenko *et al*., 2008; Deplanche *et* *al*., 2010, 2014) and also intracellularly (Omajali *et al*., 2015; Gomez‐Bolivar *et al*., 2019), while killed cells make negligible Pd‐NPs (Mikheenko *et al*., 2008; Deplanche *et al*., 2010). An important factor for sustainability, some bio‐NPs are promising heterogeneous catalysts for remediation and ‘green chemistry’ (Deplanche e*t al*., 2011; De Corte *et al*., 2012; Hennebel *et al*., 2012; Singh, 2015). They can be used in repeated cycles (Bennett *et al*., 2013) and also, self‐immobilized upon a biofilm, in continuous‐flow processes (Beauregard *et al*., 2010). NP size, shape, structure and distribution affect activity and selectivity (Lee *et al*., 2009; Schmidt *et al*., 2009). Hence, a method of altering bio‐NP formation could steer the properties of the resulting catalyst in complement to emergent methods using bioengineered microorganisms for an orthogonal approach towards ‘catalyst by design’ for specific applications.

In Gram‐negative bacteria, a population of Pd‐NPs is located in or near bacterial cell membranes and also within the periplasmic space (e.g. Mikheenko *et al*., 2008; Deplanche *et al*., 2010). Conditions which are known to change the structure or function of the cell membrane(s) and/or associated enzymes may impact upon the size, distribution, shape or hydrophobicity of the metallic NPs. A role for bacterial hydrogenases in bio‐Pd‐NP synthesis was shown using mutants deficient in one or more hydrogenase enzymes; such alterations, affecting the patterning of Pd‐NPs (Mikheenko *et al*., 2008; Deplanche *et al*., 2010), could also enhance the catalytic activity as shown in Cr(VI) reduction (Rousset *et al*., 2006).

Biogenesis of Pd‐NPs typically (but not exclusively) uses bacteria expressing hydrogenase(s), which oxidize H_2_ to 2H^+^ + 2*^e^* ^‐^, with the electrons reducing Pd(II) to Pd(0) which becomes localized as Pd‐NPs nearby (Mikheenko *et al*., 2008; Deplanche *et al*., 2010). To make ‘bio‐Pd’ catalyst, resting cells are suspended in a solution of Pd(II) ions which coordinate to ligands in/on the bacterial surface layers. The Pd(II) is trafficked to the site of its reduction by an unknown mechanism which may also relate to Ni(II) recognition and transport (discussions in Omajali *et al*., 2015; Torgeman, 2017). With an added electron donor (H_2_ or formate), the Pd(II) is reduced to metallic nanoclusters which remain patterned onto the supporting biomatrix to grow into larger NPs of size as moderated by the amount of Pd(II) supplied.

Other mechanisms of Pd‐NP biosynthesis are reported. Aerobically grown cells (i.e. not expressing hydrogenases) of *E. coli* (Foulkes *et al*., 2011) and a *Serratia* sp. (Deplanche *et al*., 2014) made catalytically active bio‐Pd. In Gram positive‐*Bacillus subtilis,* extracellular nitrate reductase was suggested to catalyse the formation of silver nanoparticles (Saifuddin *et al*., 2009) while other Gram‐positive, as well as Gram‐negative bacteria, made bio‐Pd nanoparticles with good catalytic activity in a comparative study against an industrial standard (Deplanche *et al*., 2014).

However, the catalytic activity of bio‐Pd can differ even within a single species. For example, a threefold difference was observed in soybean oil hydrogenation by bio‐Pd of two strains of *Desulfovibrio desulfuricans*, attributed to a moderation of Pd(II)‐cell interactions via different cell surface coordinating ligands and Pd‐NP patterning by cell surface bio‐polymers (Omajali, 2015). A ’bridging’ contribution of Gram‐negative cell surface lipid material was suggested by the ability of bio‐Pd to access and dechlorinate highly hydrophobic polychlorinated biphenyls (Redwood *et al*., 2008), while other work established the ability of bio‐Pd to hydrogenate hydrophobic soybean oil (Omajali, 2015; Zhu *et al*., 2016) as well as 2‐pentyne (Bennett *et* *al*., 2010, 2013), which forms the focus of this study.

For application, it is convenient to use acetone‐washed (i.e. permeabilized [Jamur and Oliver, 2010]), dried, catalyst preparations. These showed good activity and selectivity in the hydrogenation of 2‐pentyne (Bennett *et al*., 2010) soybean oil (Zhu *et al*., 2016) and itaconic acid (methylene succinate (Creamer *et al*., 2007)).

While hydrogenase mutants could produce NPs with higher catalytic efficacy (above), a biogenic catalyst must be markedly better than what is commercially available and/or be more economic to produce in order to impact upon well‐established markets (Catalytic Technology Management Ltd; commissioned consultancy report, 2009).

The goal of this study is to establish the scope for using electromagnetic intervention as a tool to produce bio‐NPs with enhanced catalytic properties, as a first step towards ‘steering’ bio‐NPs targeted towards specific reaction outcomes, in this case selectivity to the *cis*‐ene product of 2‐pentyne hydrogenation.

Radio frequency (RF) electric fields cover frequencies of 3 kHz to 300 GHz and, in some cases, they are known to affect cell membranes, e.g. such irradiation may be used for non‐thermal bacterial inactivation (Geveke and Brunkhorst, 2008). The voltage formed across the membrane in the presence of an electric field is thought to cause membrane‐thinning, electroporation and, eventually, cell rupture (Zimmerman *et al*., 1974, 1986; Hulshleger *et al*., 1981) but, in practice, it is difficult to attribute cellular damage to the electromagnetic field decoupled from local heating effects. Addressing this, Shamis *et al*. (2011) confirmed that microwave (MW) radiation (a subset of the RF range occupying the higher frequencies at 300 MHz to tens of GHz) produced a transient response in bacteria when the effects of heating were discounted. They confirmed an increased porosity in the membrane of *E*. *coli* when exposed to MW radiation at a frequency of 18 GHz and an electric field, with the temperature maintained below 40°C. Efflux of cytosolic contents was observed, and also solute penetration of cells, with increased influx, attributed to transient pores created during RF injury.

Although the application of radiofrequency (RF) electromagnetic radiation has been suggested to be able to change the structure and properties of matter, it is not usually considered for samples with a high water content due to dielectric loss. However, inorganic, dried or deep‐frozen materials can be examined at high RF strengths and powers (dielectric processing) which are known to promote alterations in crystal and surface structures. RF electromagnetic radiation at a lower dose has been applied to nanoparticle production in solution (including in culture supernatants of *Bacillus subtilis* (Saifuddin *et al*., 2009)) via thermal effects (Horikoshi and Serpone, 2013; Zhu and Chen, 2014). The radiation heats the sample via dielectric loss, converting the radiation energy into thermal energy. The two approaches that can be used i.e. (i) RF processing of dried catalyst, and (ii) of NP biosynthesis from soluble metal species within aqueous solution, produce two aims.

The first aim is to compare the effect of application of RF radiation on commercial 5 wt% Pd on carbon catalyst with dried 5wt% bio‐Pd catalyst, with respect to their catalytic activity and selectivity in the hydrogenation of 2‐pentyne. The second aim is to evaluate the scope for intervention by applying a lower RF dose during catalyst synthesis by cell suspensions, and also before exposure to Pd(II), the latter to investigate the response of the cells to RF injury with respect to their ability to make an altered Pd catalyst subsequently (‘memory’). The hypothesis is that such an intervention in cellular processes could predispose the cells to making altered Pd(0)‐NPs showing increased reaction rates and/or selectivities via altered NP localization and/or dispersions which were reported in cells of *D*. *desulfuricans* and *E*. *coli* exposed to an RF field before exposure to Pd(II) (Gomez‐Bolivar *et al*., 2019).

Towards evaluating the possible scope for physiological intervention (cell injury) within the second aim, this study used radiofrequency (RF) magnetic fields that were isolated from the heating effects produced by electric fields, via use of bespoke equipment which allows the application of RF fields to cell suspensions while allowing isolation of the thermal component to minimize thermal effects on the cells. A caveat is that Pd(II) exposure is optimally done at pH ~2–2.3 in order to protonate cell surface ligands and facilitate access of the PdCl_3_^‐^ ion (that predominates in solution) to, for example, amine ligands (Omajali, 2015). This imposes a ‘background’ dual stress (metal plus pH‐stress) on the cells, and cellular responses to both stresses have been the focus of widespread study by many authors. Delineating the combined effects of multiple stresses is beyond this scoping study which aims to show ‘cause and effect’ and hence establish the scope for RF injury as an interventional tool to underpin future applications. To achieve this, cells were exposed to an RF field at various stages of bio‐Pd synthesis, which has not been previously addressed. The bio‐Pds were evaluated in the hydrogenation of 2‐pentyne, emphasizing production of *cis‐2‐pentene*. Commercial hydrogenations are a very large industrial sector; the *cis*‐alkene is favoured over the *trans*‐isomer for a number of reasons and hence catalytic selectivity is of paramount importance (see Zhu *et al*., 2016); this also minimizes the consumption of starting material (petrochemically derived pentyne) and waste, hence generating product more sustainably.

## Results

### RF delivery via decoupled electric and magnetic fields to minimize thermal effects

**Fig. 1:**
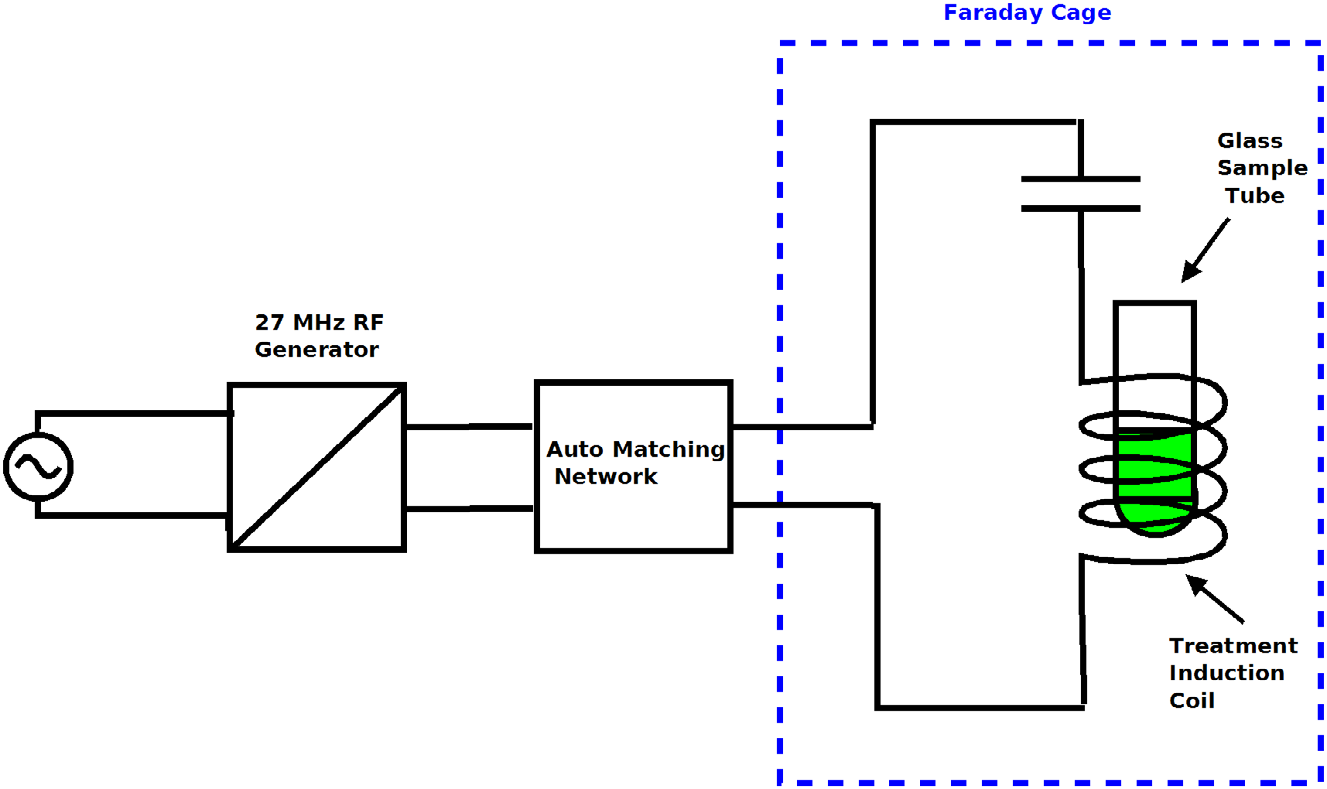
RF equipment used in this study. A Faraday cage was used to separate the magnetic field from the electrical heating components. The *E* (electric) component of electromagnetic radiation produces heating in dielectric (non‐conducting) materials; hence, it is important to exclude heating from the sample by isolating the *H* (magnetic) component. This used a series LC circuit tuned with an external matching box to generate a magnetic field inside the induction coil and an electric field in the air gap between the two conducting plates (C). The sample vial, placed in the centre of the induction coil, was exposed to the magnetic field. The samples were treated by exposure inside a solenoid coil as the electromagnetic field within the coil will be almost entirely magnetic. Consequently, as the magnetic susceptibilities of the components of the sample being treated (glass, water, bacteria and a very low concentration of Pd) are very low, at this frequency, there would be minimal heating of the sample. After exposure, the final external temperature of the bottle was checked. The temperature of the sample was monitored using a fibre optic temperature probe. Samples were treated at a power output levels of 2–60 Watts for up to 60 min as stated in individual experiments. These power levels are regarded as levels of magnetic field intensity and are proportional to the magnetic field strength but cannot be regarded as fully quantitative power densities. Samples received a dose of radio frequency magnetic field determined by the power level and the treatment time, but again, this cannot be fully quantified and data should be regarded as a semi‐quantitative for comparison.

The equipment used for RF treatment of cells and catalysts was developed in house at C‐Tech Innovation Ltd. via adaptation of commercial equipment (Fig. 1). Some sample heating (to above 30°C) was observed using a power of 8W or higher, and with exposure times in excess of 20 min. The most likely mechanisms for this are radiation or conduction of heat across the air gap from the coil (which is subject to some resistive heating). An empty glass vial showed similar heating to a full sample vial. For already‐prepared, dried bio‐Pd catalyst (and the commercial comparator), a higher power (60 W) was used. Such dried bio‐Pd was used previously in hydrogenation (Zhu *et al*., 2016) and in the Heck synthesis at temperatures in excess of 100°C (Bennett *et al*., 2013), showing thermal stability. Where living cells were to be RF‐treated before or during bio‐Pd formation, a lower exposure was used to minimize thermal effects. At a power of 8W, a temperature increase of between 2 and 6°C was observed in the sample after 20 min. Therefore, these experiments were restricted to 8W or less (usually 2–4 W; see below) for a maximum of 20 min in order to avoid thermal damage or stress to the bacteria.

### Comparison of commercial catalyst and dried bio‐Pd catalyst without and following RF treatment

**Fig. 2:**
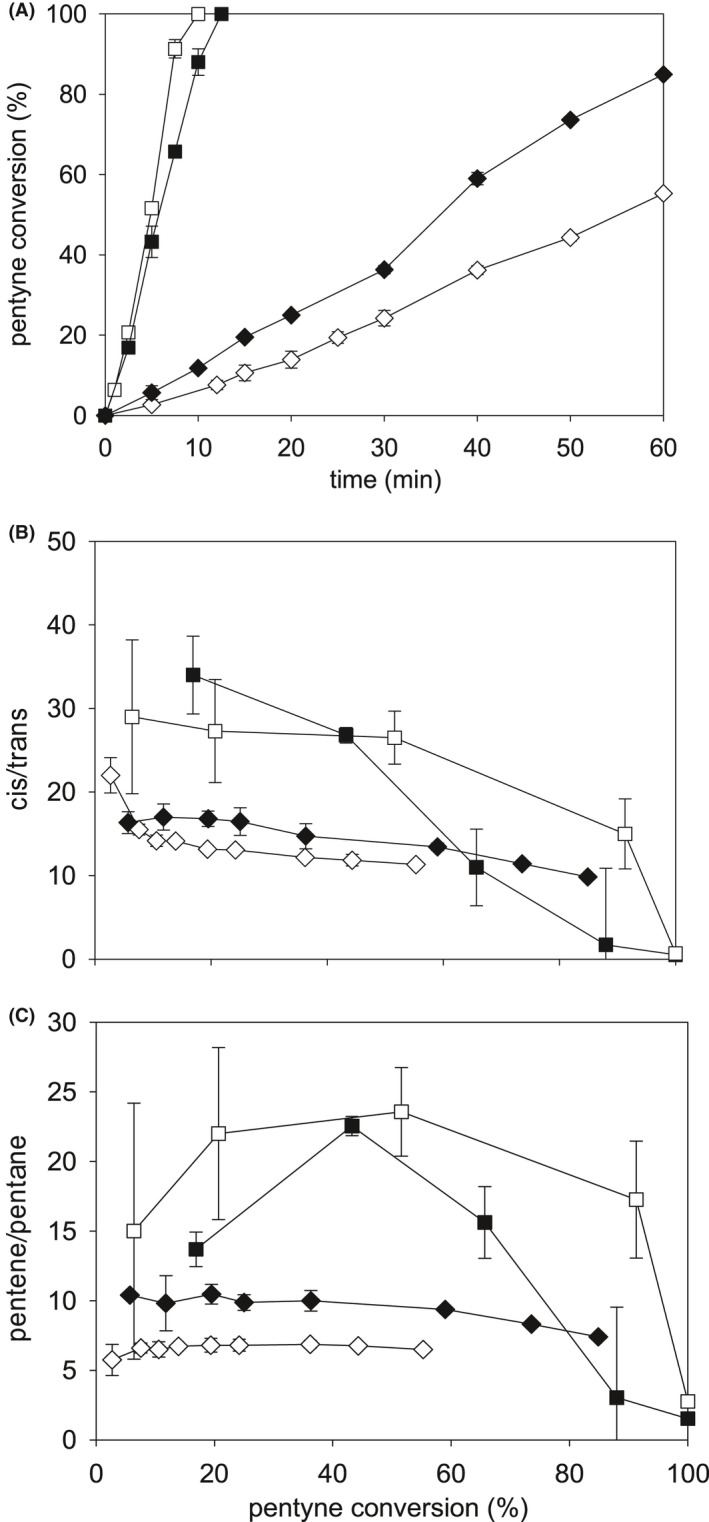
Conversion of 2‐pentyne and product selectivities using 5 wt% bio‐Pd and commercial Pd/C catalysts (open symbols) and their RF‐treated analogues (60 W; 60 min; filled symbols). A: Pentyne conversion (%). B: *Cis/trans* selectivity of pentene. C: Pentene/pentane selectivity. ■, □: 5% Pd/C. ♦, ◊: 5% bio‐Pd. Data are means ± SEM. Where no error bars are shown, these were within the dimensions of the symbols. Data were from two independent cultures with at least two technical replicates.

An earlier study compared commercial 5 wt% Pd/TiO_2_ catalyst with dried bio‐Pd (Zhu *et al*., 2016). The reaction rate was proportional to the Pd content (Zhu, 2014, 2014), and 5 wt%Pd was used in this work in order to facilitate comparison with other published work. The use of TiO_2_ was avoided in this study (to avoid solid metallic components other than Pd) and the comparator was 5 wt% Pd on carbon catalyst, as used in the hydrogenation of itaconic acid reported previously using bio‐Pd of *Desulfovibrio desulfuricans* (Creamer *et al*., 2007) and *D*. *fructosovorans* (I. Mikheenko, unpublished work; Fig. S1). In both studies, the commercial 5 wt% Pd/C catalyst outperformed bio‐Pd. In the hydrogenation of itaconic acid by the latter, the conversion rate using bio‐Pd was ~ 30% of that of the commercial comparator; in addition, the commercial catalyst achieved 90% conversion whereas the bio‐Pd achieved only ~ 70% conversion after 60 min (Fig. S1). However, the rate of 2‐pentyne hydrogenation by bio‐Pd was less than 10% of that of the commercial comparator (Fig. 2A) and with lower selectivity to *cis*‐pentene (Fig. 2B, C). Mutants of *D. fructosovorans* deficient in their Pd(II)‐reducing periplasmic hydrogenases (Mikheenko *et al*., 2008) showed the extent of itaconic acid conversion by bio‐Pd to become less than the bio‐Pd of the parent cells (Fig. S1). Other work has shown that the efficacy of bio‐Pd catalyst depends on the *Desulfovibrio* strain used for its synthesis, even within a single species: *D*. *desulfuricans* (Omajali, 2015). *D*. *desulfuricans* NCIMB 8307 was used in this work to facilitate comparison with earlier hydrogenation studies using 2‐pentyne (Bennett *et al*., 2010; Zhu *et al*., 2016).

Dried catalyst samples were exposed to RF of 60 W for 60 min (Fig. 2A). Here, the subsequent reaction rate in hydrogenation with commercial Pd/C catalyst was ~ eightfold higher than with the bio‐Pd (Fig. 2A). The rate was little‐affected by RF treatment using commercial catalyst whereas that of the bio‐Pd was increased by ~ 70% (Fig. 2A). A thermogravimetric analysis of (non‐RF‐treated) catalyst samples showed that at temperatures between 0 and 100°C, ~ 5–10% of the weight was lost, corresponding to associated water, with no additional loss of mass up to 300°C (Omajali *et al*., 2017). The samples in this study, weighed before and after treatment, showed no change in mass, i.e. biological material and bound water were not lost. The RF‐induced changes cannot be metabolic since the palladized cells were air‐dried and acetone‐treated prior to RF exposure and hence the effect of RF would reflect changes in the structure of the composite biomaterial. Microwave electromagnetic radiation at high dose is used to sinter materials (Louzguine‐Luzgin *et al*., 2009; Prette *et al*., 2011; Singh *et al*., 2015) while application of MW energy is a standard method to alter crystal structure and also has been used in the synthesis of nanoparticles via thermal effects Horikoshi and Serpone, 2013; Zhu and Chen, 2014). The temperatures of the dry samples reached a maximum of 36°C after 60 min RF at 60W, and hence, heat‐mediated structural changes were ruled out.

RF treatment had minimal effect on the selectivity of the 5 wt% Pd/C commercial catalyst up to 40% conversion, but beyond this, the treatment promoted a marked decrease in selectivity towards *cis*‐2‐pentene (Fig. 2B) and in pentene to pentane (Fig. 2C). The selectivity of bio‐Pd towards 2‐pentene was generally lower than for the 5 wt %Pd/C comparator (Fig. 2B, C), but the RF treatment increased its selectivity by a small, constant amount which was maintained at high conversion efficiencies (above 80%) where the activity of the commercial comparator became negligible (Fig. 2B, C). Hence, although the bio‐Pd catalyst was, overall, less effective in the conversion of 2‐pentyne, at a high conversion, the RF‐treated bio‐Pd shows potential advantages in maintaining selectivity. It should be noted that since the reaction outcome via the bio‐Pd and comparator catalysts is, to some extent, reaction specific (with respect to the disparity between them: Fig. S1; Fig. 2A), future evaluations should be made on a case by case basis.

### Comparison of commercial catalyst with bio‐Pd catalyst made in the presence of RF radiation

Using untreated cells, the 5 wt% bio‐Pd gave an initial rate of 2‐pentyne consumption of 1 μmol litre^‐1^ s^‐1^. A bio‐Pd catalyst prepared identically but at 40°C without RF treatment gave the same reaction rate, indicating that below this temperature, observed changes were not attributable to thermal effects. Use of 20 min exposure gave a temperature of between 36 and 40°C in the samples (aqueous suspensions). Controls established that heat‐killed cells did not make Pd‐NPs without or with RF radiation, while RF treatment of Pd (II) solution (with all ingredients except bacterial cells) did not result in reduction of Pd(II) or NP formation.

**Fig. 3:**
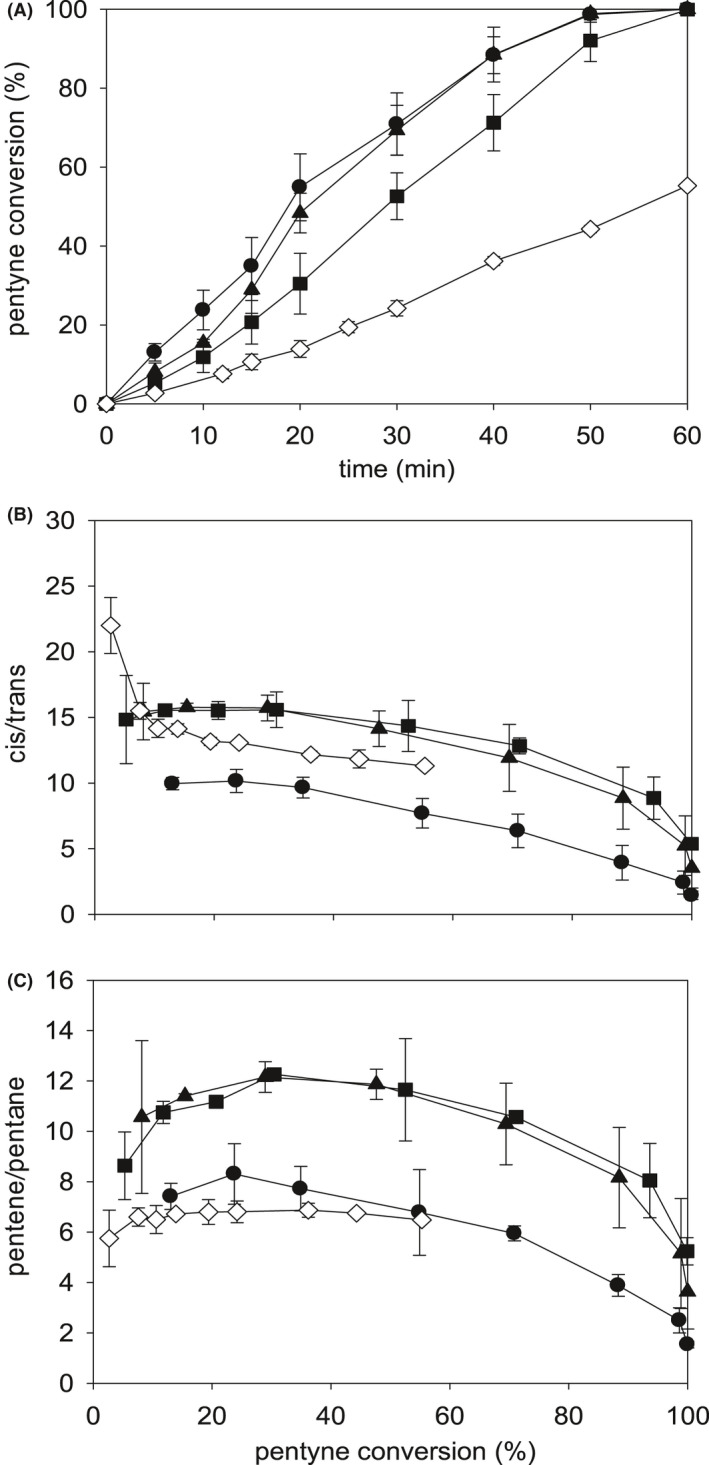
Conversions and product selectivities for cells manufacturing bio‐Pd (5 wt%) without RF treatment (control, ◊) or treated with RF (8W) as follows: ●, resting cells before addition of Pd(II); ▲, during sorption of Pd(II); ■, during reduction of Pd(II) following sorption. A: Pentyne conversion (%). B: *Cis/trans* selectivity of pentene. C: Pentene/pentane selectivity. Data are means ± SEM. Where no error bars are shown these were within the dimensions of the symbols. Data were from several pooled cultures divided for each test, with at least two technical replicates.

A power of 8W was initially selected to determine the effects of RF applied during bio‐Pd biomanufacture with respect to its subsequent catalytic activity. The RF was applied during Pd(II) uptake or during the subsequent reduction step where Pd(0) is formed. Applying the RF immediately after addition of Pd(II) solution allows the RF to take effect during uptake of the metal onto cellular nucleation sites. Application of RF after uptake of Pd(II) along with sodium formate addition allows the RF to take effect during reduction of the metal to Pd(0) at its nucleation sites. Fig. 3 shows that RF (8W) applied to cells taking up Pd(II) (and also to those exposed to RF before Pd(II) exposure‐ see later) was the most effective in terms of increased reaction rate via the resulting bio‐Pd, which was increased by more than twofold as compared to untreated controls (Fig. 3A). The advantage in selectivity towards *cis*‐2‐pentene was negated by treatment of resting cells at this dose (Fig. 3B) but was unaffected with respect to pentene selectivity over pentane (Fig. 3C). For catalysts prepared with bacteria exposed to RF during the Pd(II) uptake stage, the selectivity of the bio‐Pd was higher and was similar to where RF was applied during Pd(II) reduction only (Fig. 3B, C). The data are summarized in Table 1. This shows that the advantages in reaction rate and *cis*‐selectivity over untreated cells were the same regardless of where the RF was applied in the process of bio‐Pd biomanufacture; both treatments enriched the final mixture with respect to *cis*‐pentene (Fig. 3C; Table 1), while selectivity to pentene over the fully hydrogenated pentane was nearly doubled as compared to the bio‐Pd made by untreated cells. This choice of method was based on the maximum reaction rate coupled with the maximum selectivity, i.e. 8W RF applied during Pd(II) exposure (Fig. 3A, B). Since injury sustained prior to Pd exposure (above) resulted in enhanced catalytic activity and selectivity of the catalyst made subsequently, this was examined further.

**Table 1:** Effect of RF treatment on bio‐Pd catalyst made when dosed at 8W RF for 20 min.

Treatment during bio‐Pd(0) synthesis	Initial rate^a^ (µmol ^‐1^l^‐1^s^‐1^)	Ave *cis/trans* 2‐pentene	Ave Pentene/pentane
No RF applied	1.1*	12.8^#^	6.7^#^
RF during Pd(II) uptake	2.2*	15.2^#^	11.8^#^
RF during Pd(II) reduction	1.9^ǂ^	15.3^#^	11.5

^a^Initial rate obtained by bio‐Pd(0) in hydrogenation of 2‐pentyne up to 20% conversion. Experiments were done twice and plotted as regression lines for each experiment with errors from the mean of the gradients being as follows: *less than 5%; ^#^5–10%; ^ǂ^10–20%. With no assignation shown, the error was more than 20%. Note that addition of RF during Pd(II) reduction following RF‐free Pd(II) uptake gives larger errors but that the mean data agree closely using RF field applied in both phases of Pd(0) formation.

### Effect of lower‐dose RF treatment of resting cells before exposure to Pd(II)

**Fig. 4:**
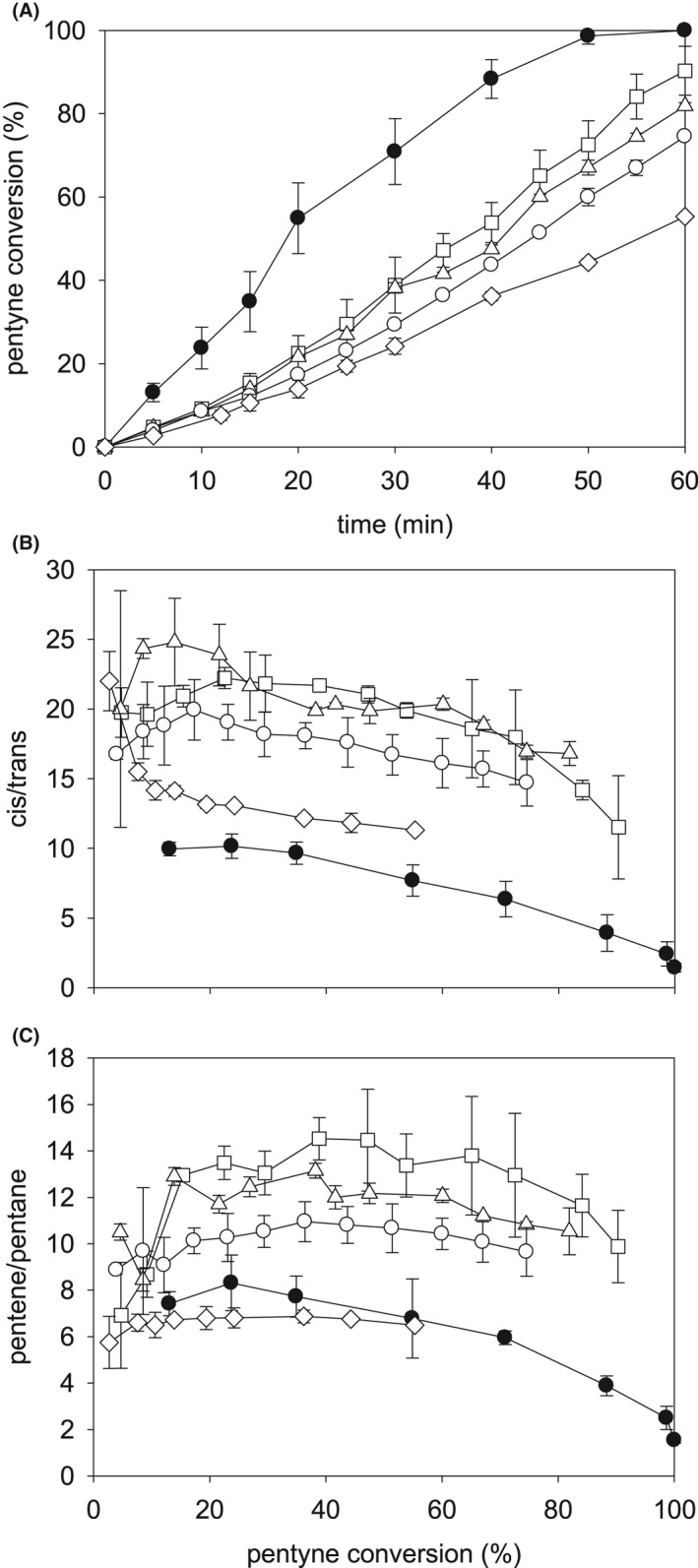
Conversions and product selectivities for bio‐Pd (5 wt% Pd) made by resting cells without exposure to RF (control, ◊) and with Pd loading following RF treatment of the resting cells as follows: ○, 2W, 5 min; ▵, 2W, 20 min; □, 4W, 20 min; ●, 8W, 20 min. A. Pentyne conversion (%). B. *Cis/trans* selectivity of pentene. C. Pentene/pentane selectivity. Data are means ± SEM. Where no error bars are shown these were within the dimensions of the symbols. Data were from several pooled cultures divided for each test, with at least two technical replicates.

To indicate a potentially altered biochemical basis for making altered Pd(0) NPs (‘memory’), the effect of 8W of RF (20 min) and lower RF doses (‘injury’) was investigated using resting cells prior to Pd(II) addition. The activities of these catalysts are shown in Fig. 4 and summarized in Table 2.

**Table 2:** Effect of RF treatment on resting cells prior to addition of Pd(II).

Treatment before bio‐Pd(0) synthesis	Initial rate^a^ (µmol l^‐1^ s^‐1^)	Ave *cis/trans* 2‐pentene	Ave Pentene/pentane
No RF resting cells	1.1*	12.8^#^	6.7^#^
5 min 2W resting cells	1.2*	18.3^#^	10.4^ǂ^
20 min 2W resting cells	1.5*	22.1*	11.8*
20 min 4W resting cells	1.6^#^	21.0^#^	12.9^#^
20 min 8W resting cells	3.3^ǂ^	9.4^ǂ^	7.6^ǂ^

^a^Initial rate obtained by bio‐Pd(0) in hydrogenation of 2‐pentyne up to 20% conversion. Experiments were done twice and plotted as regression lines for each experiment with errors from the mean of the gradients being as follows: *less than 5%; ^#^5–10%; ^ǂ^10–20%. Note that addition of RF at 8W gives larger errors, reflecting the greater cell damage shown in Fig. 5; killed cells do not form bio‐Pd(0) (see text).

A RF dose before exposure to Pd(II) improved the reaction rate via the resulting bio‐Pd in a dose‐dependent manner (Fig. 4A); the rate obtained from 4W‐pre‐treated samples was half of that of 8W‐pre‐treated samples (Table 2). Although the overall rate using bio‐Pd samples made using cells pre‐treated under milder RF conditions was lower (Fig. 4A), advantages emerged in the product selectivities, inversely related to the dose at low conversions, while at 80% conversion, little difference was seen between the treatments, with the selectivity to *cis*‐2‐pentene approaching that of the commercial catalyst (Fig. 4B; c.f. Fig. 2B).

### Visualization of MW‐injured cells

**Fig. 5:**
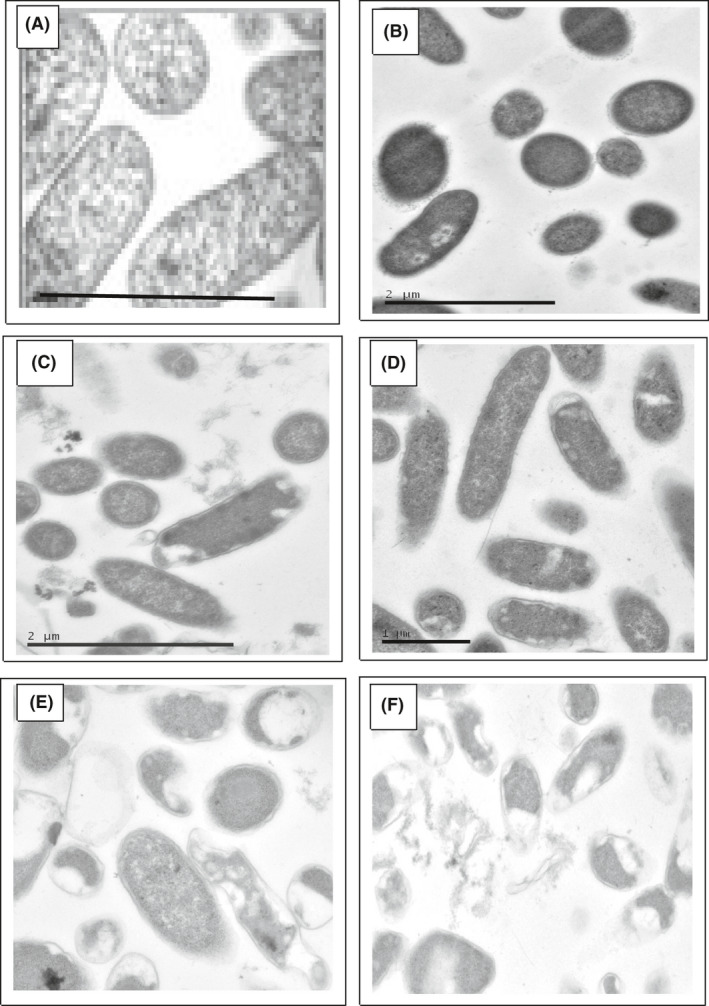
Morphological effects of RF treatment on cells of *D*. *desulfuricans*. A: Control cells without RF treatment. Cells are shown following RF treatment at B: 2W (5 min); C: 2W (20 min); D: 4W (20 min); E: 8W (20 min); F: 30W (20 min). Bars are 0.5 μm (A) and 2 μm (elsewhere). Images correspond to the data shown in Fig. 4.

Fixed samples of injured cells were examined morphologically using electron microscopy under a low accelerating voltage to minimize structural damage due to the electron beam. The images show that the cells sustained damage with increasing field power, showing vacuoles, enlargement of the periplasmic space and shrinkage of cell contents (Fig. 5). The use of markers was not used to follow leakage of cell contents, but the limit of tolerance of the cells (with respect to visible injury but no fragmentation) appeared to be 8W for 20 min (c.f. Table 3). At a dose of 15W and above (20 min; not shown), the cells were unable to reduce Pd(II), confirming that active cells are required for NP formation and ruling out RF‐mediated NP synthesis on RF‐altered cell structures. A 30W dose (20 min) revealed extensive cell destruction (Fig. 5).

A companion study (Gomez‐Bolivar *et al*., 2019) applied high‐resolution electron microscopy to examine Pd‐NPs made by untreated cells and cells treated before Pd(II) addition, but the effect of co‐applying RF and Pd(II) has not been studied previously. In complement to Fig. 5, shrinkage of the cellular compartment was confirmed in each case (Fig. S2) with the Pd‐NPs visualizing the retracted cytoplasmic membrane and enlarged periplasmic space which contained few NPs. Gomez‐Bolivar *et al*. (2019) also reported the lattice spacings (Å) of Pd‐NPs of RF‐untreated and ‐treated cells as, respectively, 0.223 and 0.231 (111 facets) and 0.199 and 0.202 nm (200 facets), indicating no major differences in the crystal structure between them. However, Omajali *et al*. (2015) noted that the crystal structure of *D*. *desulfuricans* bio‐Pd shows ~ 5–10% distortion as compared to bulk Pd(0); any RF‐promoted differences would be hard to discern, and hence, NPs made in the presence of Pd shown in Fig. S2 were not examined at high resolution due to this limitation.

Addition of RF along with Pd(II), which has not been reported previously, would allow more facile production. In each case, the NP population was more homogeneous as compared to untreated cells (unpublished work; Fig S2). Note that the NPs of untreated cells featured apparent ‘outgrowths’ (and agglomerations) that were absent from NPs of cells treated with RF by either method i.e. regardless of whether Pd was present during or following RF treatment (Fig. S3). Close inspection shows apparent ‘recruitment’ of small NPs into larger ones in control cells, and this process being negated following RF injury before or with Pd(II). It was considered that magnetic attraction between NPs may have promoted NP aggregation; ferromagnetism of bio‐Pd‐NPs was reported (Creamer *et al*., 2011; Williams, 2016). Hence, we attempted to visualize magnetic alterations of the population by scanning X‐ray imaging with image analysis (made possible by use of a polarized synchrotron radiation beam) and magnetic force microscopy to view individual cells. However, neither of these methods were successfully applied due to their low spatial resolution (Fig. S4).

## Discussion

This study reveals different outcomes of RF treatment of dried bio‐Pd catalyst and of living cells (with or without Pd(II)) with a lower RF dose and hence, we suggest, two mechanisms of catalyst activity enhancement.

Our first conclusion is that RF processing enhances the activity of dried bio‐Pd catalyst but not its commercial counterpart. This may suggest structural alterations in the supporting biomatrix/NP interface (note that no mass was lost during RF treatment: above). Direct interactions between cellular materials and Pd(0), reported previously (Priestley *et al*., 2015), were revealed by high‐resolution electron microscopy, as a ‘pancaking’ of Pd‐NPs onto the biomatrix at the bio‐metallic interface (Bennett *et al*., 2013). Differences between bio‐Pd and metallic Pd were also indicated by ^105^Pd NMR (Hooper *et al*., 2018), while electron paramagnetic resonance showed enhanced electronic interactions, to an extent which paralleled the electrocatalytic activity (Carvalho *et al*., 2009). A ‘steerable’ effect of the biomatrix is inferred, the nature of which is still unknown (see later).

The conversion rate via the RF‐treated biomaterial was less than the commercial comparator, but with advantages in selectivity at high conversions (i.e. long reaction times). An increased yield of desired product per kilo of starting material (2‐pentyne) would reduce the carbon impact, via lower consumption of petrochemically derived substrate as well as a reduction in waste unwanted by‐products. Fewer, longer reaction, times would bring economic benefits in terms of less reactor ‘down time’. Potential advantages should be evaluated on a case by case basis, e.g. between well‐established large‐scale hydrogenation operations as compared to other, possibly niche, applications and/or where the cost/carbon impact of the starting material, disposal of wastes and ease of production are major considerations; the importance of these parameters is evolving during the transition from fossil fuels into lower carbon processes.

The Pd‐bionanoparticles, like chemically made Pd‐NPs, contain Pd‐(111) facets, are icosahedral (Bennett *et al*., 2013; Omajali *et al*., 2015), and they are supported (stabilized) on the (hydrated) biomatrix. The biomaterial was dried in air but bound water in (e.g.) hydrated polymers remains (deduced from thermogravimetric analysis: Omajali *et al*., 2017). Local perturbations in bound water molecules could result in morphological rearrangement of the biochemical/metal interface which was not investigated here. However, normally water absorption is an electric field effect whereas the field in this case was almost exclusively magnetic.

Two studies of bio‐Pd‐NPs have shown that they are ferromagnetic (Creamer *et al*., 2011; Williams, 2016), a property that is shared with chemically made Pd‐NPs within particular size boundaries (see Williams, 2016 for discussion). Pd‐NPs tend to agglomerate into larger NPs unless stabilized by capping agents. This function is provided at the biomatrix/Pd‐NP interface, where Pd atoms ‘pancaked’ at the edge of the NP (Bennett *et al*., 2013), i.e. increasing the planar surface component. Partial chemical ‘cleaning’ of the biomaterial (bio‐Pt in this case) enhanced reaction selectivity (Bennett *et al*., 2012; Attard *et al*., 2013), giving scope for post‐synthesis optimization, albeit at additional cost.

Earlier work reported that semihydrogenation of 2‐methyl‐3‐butyn‐2‐ol to 2‐methyl‐3‐butene‐2‐ol occurs preferentially at plane sites of the crystal whereas (undesired) hydrogenation of alkene to alkane occurs mainly at edge sites (Crespo‐Quesada *et al*., 2011). In addition to ‘moderating’ site availability, the presence of stabilizing biomaterial may also have conferred some protection of the NPs to fragmentation (which would otherwise make more edge sites, promoting undesirable alkane production) which occurs during energetic treatment of crystals (Collins and Bettens, 2015).

Teschner *et al*. (2006) reported that selectivity in pentyne hydrogenation also related to the exclusion of bulk hydrogen via the buildup of a Pd‐C layer, explaining that this buildup process is not effective on the surface of bulk (111). Hence, bio‐NPs with predominantly (111) surface planes (i.e. bio‐Pd‐NPs) might be expected to be less selective in hydrogenation because hydrogen can saturate the bulk of the particle, which becomes too reactive (i.e. a faster reaction; but note that the rate with bio‐Pd was slower: above) at the expense of selectivity. Teschner *et al*. (2006) also suggested that structural defects may be required to facilitate initiation of the Pd‐C surface phase that then retards participation of bulk‐dissolved hydrogen in the reaction, hence favouring selectivity at the expense of rate. In this mechanistic context, a more detailed comparative structural examination of the population of metallic NPs of RF‐injured cells would be warranted in future work. However, the previous studies, using electrochemical and shell‐isolated nanoparticle Raman spectroscopy (SHINERS) methods (Bennett *et al*., 2012; Attard *et al*., 2013), used bio‐Pt; electrochemical examination is less applicable to bio‐Pd since Pd(0) holds large amounts of hydrogen atoms on and in the crystal structure (see Courtney *et al*., 2016 for discussion).

The second conclusion of this work is that pre‐injury of bacteria (2 W or 4 W power) gave a faster (by ~ 50%) rate of hydrogenation via the resulting bio‐Pd catalyst than that shown by untreated bacteria, as well as > 50% increase in selectivity. It must be noted that commercial catalysts have been extensively developed and optimized prior to market whereas this is the first study of its kind using bio‐Pd and with no attempt made at bio‐catalyst optimization nor comparative application in different reactions. For example, Zhu *et al*. (2016) showed that bio‐Pd outperformed a commercial catalyst (Pd/TiO_2_) in the selective hydrogenation of soybean oil, while Deplanche *et al*. (2014) showed comparable activity of this bio‐Pd in the Heck synthesis in an industrial laboratory validation against commercial comparators used routinely for that reaction. However, different catalysts may have different, case‐specific optimal Pd loadings. For example, Fig. S1 (unpublished) shows variation in three catalysts in the hydrogenation of itaconic acid (using parent cells and mutants of a single organism: *D*. *fructosovorans;* bio‐Pd catalytic activity can differ between *Desulfovibrio* strains‐ – see earlier). The % conversions (at 30 min) for cells of *D. fructosovorans* loaded with Pd (wt%) of 10%, 5% and 2% were 50.0 ± 1.84, 55.25 ± 3.11 and 42.06 ± 4.95 (I. Mikheenko, unpublished), while Zhu (2014, 2014) reported that with *D. desulfuricans* bio‐Pd (compared with 5wt% Pd on TiO_2_), the rates for 5wt% and 2wt% bio‐Pd were correspondingly 5:2.

A stringent comparison is only realistic under the optimal Pd loading and reaction conditions for each reaction, compared against the best commercial catalyst for each, which were not attempted. Indeed, two independent commercial catalysts gave different results when compared to bio‐Pd (Creamer *et al*., 2007; Zhu *et al*., 2016) and hence a ‘like for like’ comparison is not trivial. In this study, 5 wt% Pd on carbon was chosen as the comparator, to facilitate comparison with hydrogenation of itaconic acid (Creamer *et al*., 2007) but mainly because the use of additional metallic supports or components should be avoided in order to preclude deflection of the RF field and the formation of localized ‘hot spots’, to ensure a homogeneous field in the sample chamber. In illustration, while the same results were obtained using cells RF treated before and during bio‐Pd synthesis, the errors were larger in the latter (Table 3), which may indicate inhomogeneities at the nanoscale, i.e. via metallic Pd‐NPs inducing local RF field perturbations upon their initiation and onward growth, but this was not examined further.

The beneficial effect of a low RF dose on living cells before Pd(II) exposure (as well as when added along with Pd(II)) indicates that formation of improved catalyst is rooted in a biological response to RF injury. Dynamic studies using flow cytometry were not possible due to separate locations of the RF apparatus and flow cytometry equipment; microwave‐injured cells showed recovery in about 10 min (Shamis *et al*., 2011). Visualization of *D*. *desulfuricans* cells by electron microscopy (immediately‐ fixed cells: Fig. 5) shows a similar morphological response to that described by Shamis *et al*. (2011) in terms of retraction of the cytoplasmic membrane, shrinkage of the cellular compartment and enlargement of the periplasmic space, which was confirmed at higher resolution in a related study which visualized deposited Pd(0) (Gomez‐Bolivar *et al*.,^2019^); here, the pre‐injury resulted in Pd internalization to the inner membrane, not seen in untreated cells. The deposited Pd(0) can be regarded as a ‘fossil record’ of the altered uptake pattern of Pd(II), attributed to transient breakdown of the outer membrane and altered flow patterns across it.

Williams (2016) showed by magnetic analysis (bulk population), and direct examination (EM of individual cells), three subpopulations of bio‐Pd‐NPs of different sizes, has confirmed in pre‐injured cells (Gomez‐Bolivar *et al*., 2019) and in cells RF treated with RF in the presence of Pd(II) and during its reduction (unpublished; Fig. S2). Pd‐particle size, as well as topography, is known to affect the reactivity in olefin hydrogenation/isomerization (Lee *et al*., 2009; Schmidt *et al*., 2009); these features may not be offered identically by component NPs in each subpopulation and hence an RF‐injury‐induced change in NP size distribution may reflect an altered ‘expression’ of different NP proportions, attributable to different proportions of the active hydrogenases in response to metabolic disruption (c.f. Fig. S1).

Untreated cells had a sixfold greater predominance of smaller NPs than RF‐treated cells which was reduced to a threefold excess with cells injured before or during Pd(II) addition, i.e. greater homogeneity was promoted by RF injury (i.e. a reduced dispersivity index). Figure S2 shows, for comparison, unpublished data for cells RF‐challenged in the presence of Pd(II), while Fig. S3 reveals that, while the component of larger NPs of untreated cells comprised aggregations of smaller ones, their absence in the cells treated before, or in the presence of Pd(II), suggests that a biochemical factor predisposes the cells to form NP aggregations which may be ascribed to mutual interactions (possibly magnetic) of the NPs of uninjured cells which are lost as an effect of RF treatment. The loss of this aggregation may suggest that the difference in the NPs is rooted within the biochemical functionalities (e.g. participating hydrogenases) affected by the RF field. Bio‐Pd ferromagnetism (see earlier; Williams, 2016) is an electron spin‐related phenomenon; hydrogenase can flip the electron spin (of exiting electrons) *via* redox active metals at the active site during hydrogen splitting **(**Rousset *et al*., 1998; De Lacey *et al*., 2007). In accordance with the localization of Pd(0) NPs to ‘tag’ hydrogenase activity (via capture of the emitted electron stream into Pd(II) reduction (Mikheenko *et al*., 2008)), stable spin‐polarized electrons on bio‐Pd were visualized by direct injection (as a ‘surrogate’ of hydrogenase) of spin‐polarized electrons into bio‐Pd (Mikheenko *et al*. 2012 **)**. It follows that a Pd acting as a ‘spin trap’ would report on the ‘health’ of the responsible hydrogenase. This assumes activity of formate hydrogen lyase in *Desulfovibrio* to generate H_2_ from formate (Matins *et al*., 2016), the electron donor supplied here (to avoid the spark‐hazard of gaseous H_2_). Attempts to visualize and quantify the magnetic features of the bio‐Pd were precluded by the low resolution of available technologies (Fig. S4). Attribution of altered Pd(0) NPs as a result of altered hydrogenase(s) activity via cell injury remains speculative, and hence, further (non‐trivial) studies are required to probe the magnetic and spin state of the bio‐Pd made by RF‐injured cells. The spin polarization on the bio‐Pd‐NP surface is stable (Mikheenko *et al*. 2012) and warrants further study: the role of electron spin in selective catalysis is now established; for example, a recent review by Naamen *et al*. (2019) describes the enantiospecific interaction between ferromagnetic substrates and chiral molecules, while Metzger *et al*. (2020) report a role for spin‐polarized electrons in achieving reaction selectivity. These authors propose this as a new mechanism to realize enantioselective chemistry; hence, we tentatively suggest that bio‐Pd can contribute this functionality via ‘feeding’ spin‐polarized electrons into developing bio‐Pd‐NPs (via hydrogenase) that surpasses ‘classical’ Pd‐NPs and is not achievable by chemistry alone. Alterations in participating hydrogenase proportions (and their activities) may influence the spin‐polarized components on the resulting bio‐Pd‐NP surface. The present case may infer that ‘normally’ spin polarization would produce the undesired *trans*‐isomer since *cis*‐selectivity was improved under RF treatment. This introduces the concept of manipulating the spin polarized component via altered hydrogenase activity(ies). This remains to be confirmed, e.g. the RF effect may not rest only on the hydrogenase(s) but also on metabolic processes within which they operate (see below).

A related study (Gomez‐Bolivar *et al*., 2019) used high‐resolution elemental mapping to show more Pd internalization within *E. coli* cells following RF treatment, suggesting enhancement of cellular permeability under RF stress as proposed by Shamis *et al*. (2011). Cellular Pd uptake was indicated by Pd localization at a nuclear body (Omajali *et al*., 2015) while close inspection of the cells without RF and with RF treatment during Pd exposure confirms some small NPs within them (Fig. S2).

The means by which Pd(II) ions are assimilated is currently unknown but, since bacteria have well‐defined metal trafficking pathways (uptake and also efflux) for essential metals which require homeostasis to avoid toxicity, an association with the metal‐transport and assimilation/efflux pathway(s) may be anticipated. For example, Pd(II) may be ‘recognized’ in lieu of, for example, Ni(II) (see Omajali *et al*., 2015 for discussion). Hydrogenases and other enzymes contain Ni, which gives a number of potential ‘destination’ sites invoking cellular ‘recognition’ of Pd(II), but an inability to produce a functional enzyme that is dependent on the precise incorporation (and redox potential) of the metal required for enzymatic activity. The low content of intracellular Pd‐NPs in uninjured *D*. *desulfuricans* (above) may relate to an effective efflux mechanism for excess Pd(II) ions with metal reduction occurring also during exit from the cells. No attempt was made to differentiate between NPs made during Pd(II) uptake or egress; this has been hampered by available methodologies.

A rapid method to visualize altered Pd accumulation Torgeman (2017) screened 21 d‐block metal transporter gene mutants of *E*. *coli*, showing influential effects of several genes relating to transport/efflux of copper, silver, cobalt, molybdenum, and iron(II). Unpublished work examining the effect of RF exposure on gene expression in *E*. *coli* showed a three‐ to fourfold upregulation of a suite of genes including functionalities relating to Ni(II) metabolism (Fig. S5). Indeed, prior to the work we report here, it was known that exposure of cells to an electric field influences various cellular processes (Knowles *et al*., 2007), while an electric field was shown to enhance hydrogenase‐mediated hydrogen production by *E. coli* (Redwood, 2007). However, these unrelated studies did not attempt to isolate electric from magnetic field components. RF‐injured cells, while showing outer membrane rearrangement and increased permeability (Shamis *et al*., 2011), apparently recovered within 10‐15 min. Altered gene expression and protein synthesis (Fig. S5) may be a secondary response triggered by the membrane perturbations and loss of permeability barrier. Both periplasmic and inner membrane‐bound hydrogenases are involved in Pd(0) deposition in *D*. *desulfuricans* (Mikheenko *et al*., 2008); it is likely that the RF magnetic field that induced changes to the membrane environment perturbed the transmembrane processes of hydrogen cycling which are key to the metabolism and health of *D*. *desulfuricans*.

Compared to cells injured in the absence of Pd, a simple increase in subsequent NP homogeneity post‐injury (Gomez‐Bolivar *et al*., 2019; Fig S3) via post‐stress injury response(s) and altered Pd‐patterning might account for the decreased (halved) rate and doubled selectivity at lower doses. It could be argued that the short transfer time between introduction of the RF field and Pd(II) addition was sufficient to enable the response to persist during Pd(II) uptake, but this cannot account for the similar effect on catalysis by bio‐Pd made by cells only exposed to RF during the reduction stage. Hence, a further, slower response may be suggested which relates to the ‘handling’ of the Pd(II) within the cellular matrix. Once the NPs were initiated, the catalytic activity was identical (within error) with respect to application of RF during Pd(II) uptake or during reduction to Pd(0); the mean NP size was larger (by about 25%) if cells were RF treated only during Pd(II) reduction (Fig S2) but larger errors were seen (Table 1) possibly attributable to local RF field perturbations during NP growth via nascent Pd‐NPs in catalysis. However, simultaneous application of RF and reductant would simplify NP synthesis and reduce production costs.

Enhanced selectivity was shown by bio‐Pd made with RF in the presence of Pd(II) (Table 1) while the NP homogeneity was greater (Fig. S2 and S3). Together these may indicate a possible change in the surface atomic arrangement (and/or defects) of the bio‐supported Pd‐NPs as well as their precise interactions with influential biochemical scaffolding matrices. Relevant to this conjecture, temperature‐programmed desorption studies of *cis*‐ and *trans*‐2‐butene over various platinum surfaces (Lee *et al*., 2009) showed that the *cis*‐isomer was more thermodynamically stable upon close‐packed Pt(111), (100) planes than the *trans*‐isomer. Conversely, the latter was more stable on a more open Pt(110); this underlines that the facets and atomic arrangements of the Pd atoms on bio‐Pd made after and/or during RF irradiation warrant a more exhaustive study.

Intuitively, larger NPs (such as those produced by *D*. *desulfuricans* under RF stress: Fig. S2) should result in a slower reaction rate for the same mass of metal (due to the relatively lower overall surface area to volume) but, on the other hand, Doyle *et* *al*. (2004, 2005) showed that pentenes react faster on larger particles; the smaller particles observed in other work following RF treatment of *E*. *coli* (Gomez‐Bolivar *et al*., 2019) are in accordance with this (i.e. reduced formation of pentane) but the opposite observation reported for *D*. *desulfuricans* (whereby the NPs become larger overall with cell injury before or during Pd addition/reduction) would anticipate more pentane (at the expense of pentene) but this was not observed (Tables 1 and 2). However, some larger NPs of native cells comprise agglomerations of smaller ones (see above discussion: Fig. S3); the protrusion of small NPs ‘standing proud’ would tend to retard pentane reactivity (above). The RF‐treated cells did not show agglomerations of NPs either with RF applied to resting cells (Gomez‐Bolivar *et al*., 2019) or during Pd(II)‐NP synthesis (unpublished work: see Fig S3).

The cellular responses leading to the formation of altered NPs, dispersions and catalytic activities are complex; over‐conjecture at this stage is premature, but altered Pd‐NPs can be regarded as providing a ‘fossil record’ of the history of such changes which cannot easily be followed dynamically.

The situation is further complicated by a recent report that the Pd species on sulfidogenic cells is not only Pd(0) but also an oxidized form (PdO; the catalyst was stored in air) as well as a sulfided form of Pd, revealed by elemental mapping (Gomez‐Bolivar *et al*., 2019) with Pd‐sulphur bonds confirmed by X‐ray photoelectron spectroscopy (Omajali, 2015; Mikheenko *et al*., 2019) (e.g. PdS, but Pd_3_S, Pd_4_S or PdxSy are also reported in the chemical literature). The cells were washed before use but the possibility of residual H_2_S production by resting cells was not precluded (Mikheenko *et al*., 2019). Although sulfide is a classical catalyst poison, Pd_4_S was reported as one of the most selective hydrogenation catalysts in the hydrogenation of alkynes to alkenes, with PdS also showing high –ene selectivity (McCue *et al*., 2016). More recent work (Albani *et al*., 2018) has reported the high activity of a nanostructured Pd_3_S phase as well as noting that ethane desorption from Pd_3_S and Pd_4_S surfaces (0.08 and 0.22 eV respectively) is energetically preferred over further hydrogenation of the ‐ene (0.79 and 0.55 eV respectively). This is the opposite situation to Pd(111) where the overhydrogenation is favoured (barrier is 0.45 eV), and this being much lower than that of the alkene desorption (0.85 eV).

In the light of these two recent developments (Albani *et al*., 2018; Mikheenko *et al*., 2019), a full crystallographic and ultrastructural examination of the Pd products of sulfidogenic cells is warranted but is complicated by the fact that the lattice spacings are very similar (see Mikheenko *et al*. (2019) for discussion). If a pivotal role for Pd‐sulfide is confirmed, then a potential advantage offered by sulfidogenic bacteria would be that the dissimilatory sulphate metabolism of the cells under RF stress may provide another interventional target for fine‐tuning. As the RF field has profound effects on membrane integrity and potentially on hydrogen cycling (earlier), the proton gradient would be affected so that ATP cannot be formed by ATP synthase. In this respect, the RF may be acting like a classical uncoupling agent and thus promote increased flux into sulphate reduction, H_2_S formation and more predominance of palladium sulfide components of the NPs. The putative Pd‐S‐NPs are under current examination.

## Experimental procedures

### RF delivery system

The bespoke system (Fig. 1) comprised a 27.5 MHz RF generator (10P Plasma Products Inc., Cherry Hill, NJ, USA), matching network to tune the circuit and an applicator circuit of a capacitor plate and a solenoid induction coil situated in a Faraday cage. The coil was of a slightly larger internal diameter than the glass sample vial, which was fully immersed in the coil and stood on a ceramic holder (Fig. 1). Test vials (glass, Wheaton) were 51 mm x 22 mm diameter (working volume 12 ml) containing 10 ml of cell suspension.

### High‐dose radiofrequency magnetic field treatment of pre‐formed palladium catalyst

Bio‐Pd catalyst (5 wt% Pd: Pd: biomass dry weight) was prepared on cells of *D*. *desulfuricans* as described previously (Bennett *et* *al*., 2010, 2013) with 0.1 M sodium formate solution here substituted for H_2_ gas for metal reduction (2.5 mmol sodium formate to 1 mmol Pd(II), left overnight). Following complete removal of Pd onto the cells (determined by assay: Bennett *et* *al*., 2010, 2013), the sample was washed in water then acetone (in order to permeabilize the cells: Canovas *et al*., 2005; Jamur and Oliver, 2010), dried and ground to form the catalyst. The comparator was commercial 5 wt% Pd/C catalyst (Creamer *et al*., 2007). A sample of dry, pre‐weighed catalyst was added to the sample tube and placed in the centre of the coil in the RF treatment system (Fig. 1). A radiofrequency magnetic field (27.5 MHz) of 60 W power was applied (60 min). The temperature of the sample was monitored throughout via a fibre optic temperature probe, and the sample was reweighed after RF exposure to check for any change of mass.

### Low‐dose RF magnetic field treatment of live cells followed by bio‐Pd synthesis

A suspension of resting cells in 20 mM MOPS–NaOH buffer (pH 7) of known dry weight ml^−1^ was added to a sealed sample tube under a N_2_ atmosphere and placed in the centre of the coil in the RF generator. A radiofrequency magnetic field (27.5 MHz; 2–60 W) was applied (5–30 min) as described in individual experiments. The sample was removed from the field, and the temperature was checked. Parallel controls were left in buffer without application of RF and allowed to make Pd(0) alongside the RF‐treated samples. Na_2_PdCl_4_ solution (2 mM in 10 mM HNO_3_; hydrogenase activity was previously shown to persist in this cell suspension for the duration (I. Mikheenko unpublished)), previously degassed and stored under N_2_, was added by syringe; the volume was that required to give 5 wt% Pd (dry mass of cells was calculated from the OD600 of the suspension). The solution was left (20 min) for the bacteria to take up the Pd(II) before adding 0.1 M sodium formate solution (electron donor: 2.5 mmol sodium formate to 1 mmol Pd(II)) and left overnight. The cells were centrifuged, washed and dried as described previously, including an acetone wash (Bennett *et* *al*., 2010, 2013).

### Examination of cells

Before or immediately following RF injury, the live cells were fixed in glutaraldehyde and dehydrated (ethanol series), sectioned and routinely examined by electron microscopy as described previously (Deplanche *et al*., 2010). Examination of ‘palladized’ cells by transmission electron microscopy was as described by Gomez‐Bolivar *et al*. (2019). Examination of populations of ‘palladized’ cells using synchrotron radiation‐based scanning X‐ray microscopy (SRSXM) to quantify co‐distributions of Pd and light elements was as described by Mikheenko *et al*. (2019). Magnetic imaging was not employed due to insufficient resolution.

### RF magnetic field treatment of live cells during bio‐Pd synthesis

To evaluate the effect of RF radiation on the processes of uptake of Pd(II) and initial Pd deposition, the resting cells (and untreated controls) were placed in the RF magnetic field with Pd(II) as above during the period of Pd(II) uptake (20 min or as stated) and removed from it before formate addition (2.5 mmol sodium formate to 1 mmol Pd(II)). Alternatively, cells were untreated during Pd(II) uptake and then transferred to the RF field during the reduction of Pd(II) to Pd(0) (20 min) and then left overnight (no field).

### Catalytic testing in the hydrogenation of 2‐pentyne

5 wt% Pd catalyst (30 mg commercial 5wt% Pd on carbon catalyst or 5 wt%bio‐Pd, i.e. 1.5 mg Pd(0) metal throughout) and 150 ml of 2‐propanol were added to a 500 ml Baskerville autoclave reactor, sealed, purged with N_2_ and stirred (500 rpm, 40°C) with hydrogen bubbled through (0.1 litre min^‐1^; 10 min) to ‘prime’ the catalyst. The reactor was purged with N_2_, and 2‐pentyne (4 ml) was added. The reactor was flushed with H_2_, sealed and pressurized (2 bar). The stirred (1000 rpm) mixture was sampled periodically; analysis used GC (Varian CP‐3380 GC with a flame ionization detector and a 25 M ChrompackPlot CP 7576 capillary column with an Al_2_O_3_/KCl coating). The oven temperature ramp was: initial temperature of 95°C (30 min) ramp to 220°C (50°C min^‐1^) and hold (20 min).

## Funding Information

EPSRC (EP/I007806/1; EP/D05768X/1), BBSRC (BB/C516128/1), NERC (NE/L014076/1), The Royal Society (Industrial Fellowship) and Spanish Government Sistema Nacional de Garantia Juvenil grant PEJ‐2014‐P‐00391.

## Conflict of interest

None declared.

## Supporting information

**Fig. S1**. Hydrogenation of itaconic acid by bio‐Pd of Desulfovibrio fructosovorans.

**Fig. S2**. Pd‐NPsproducedbyRF‐injured*D.desulfuricans*.A:noRFapplication(control)andRFappliedtorestingcellsbeforePd(II)addition(B)(Gomez‐Bolivar*etal*.,2019)orduringPd(II)uptake(C).DispersivityindexeswereA:2.07;B:1.26;C:0.98(calculatedaccordingtoGomez‐Bolivar*etal*.,2019).

**Fig. S3**. Effect of RF treatment on Pd‐NP formation.

**Fig. S4**. Magnetic imaging of cells loaded to 5wt% Pd(0).

**Fig. S5**. Mobile phone ‘communication’ with E. coli perturbs the expression of genes involved in fundamental cellular processes.
