# Supplementary material for: Enhanced hydrogenation catalyst synthesized by Desulfovibrio desulfuricans exposed to a radio frequency magnetic field
Source: Microb Biotechnol. 2021 Jul 3;14(5):2041–58. doi: 10.1111/1751-7915.13878 (PMC8449679; doi:10.1111/1751-7915.13878)
Supplement: Supplementary file 1 — Fig. S1. Hydrogenation of itaconic acid by bio‐Pd of Desulfovibrio fructosovorans. [file MBT2-14-2041-s001.pdf]

Figure S1. Hydrogenation of itaconic acid by bio-Pd of *Desulfovibrio fructosovorans*

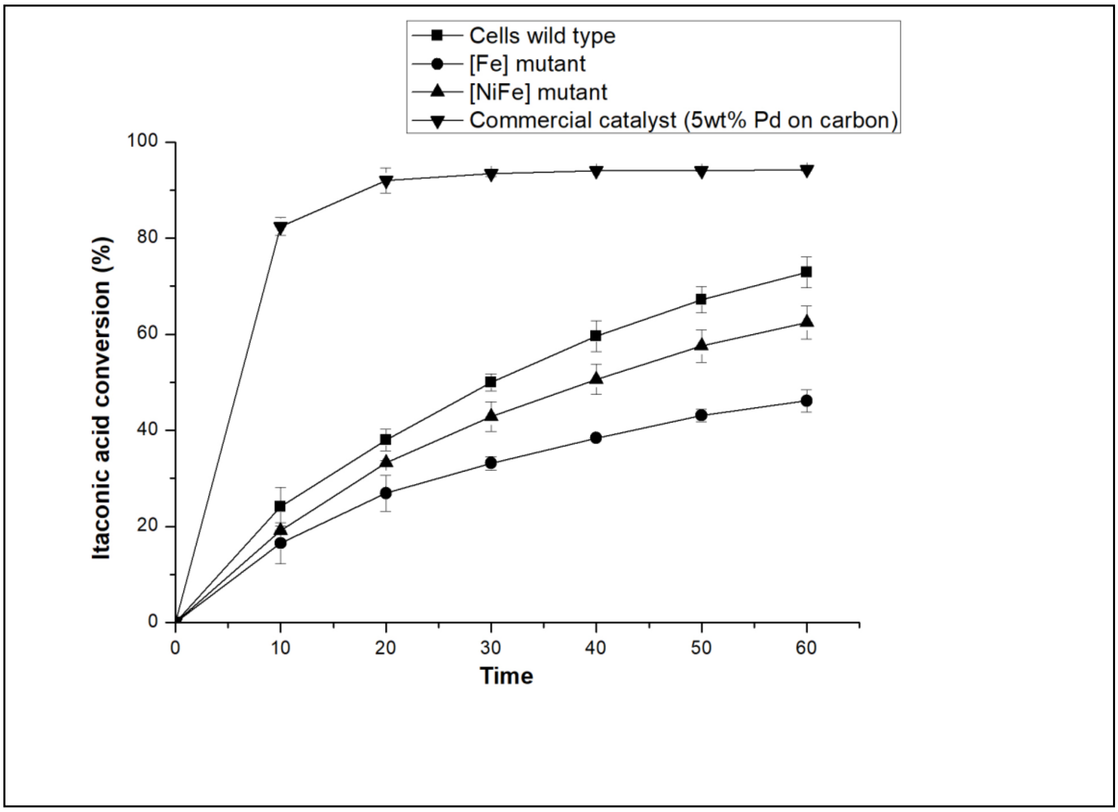

Bio-Pd (10 wt% Pd) was made on *Desulfovibrio fructosovorans* using parental cells (wild type, WT) and mutants deleted with respect to their periplasmic hydrogenases ([Fe] hydrogenase or [FeNi] hydrogenase: mutants were as described by Mikheenko *et al*. (2008). The commercial comparator was 5wt% Pd on carbon catalyst (as described by Creamer *et al*., 2007).
