# Supplementary material for: Enhanced hydrogenation catalyst synthesized by Desulfovibrio desulfuricans exposed to a radio frequency magnetic field
Source: Microb Biotechnol. 2021 Jul 3;14(5):2041–58. doi: 10.1111/1751-7915.13878 (PMC8449679; doi:10.1111/1751-7915.13878)
Supplement: Supplementary file 2 — Fig. S2. Pd‐NPsproducedbyRF‐injuredD.desulfuricans.A:noRFapplication(control)andRFappliedtorestingcellsbeforePd(II)addition(B)(Gomez‐Bolivaretal.,2019)orduringPd(II)uptake(C).DispersivityindexeswereA:2.07;B:1.26;C:0.98(calculatedaccordingtoGomez‐Bolivaretal.,2019). [file MBT2-14-2041-s002.pdf]

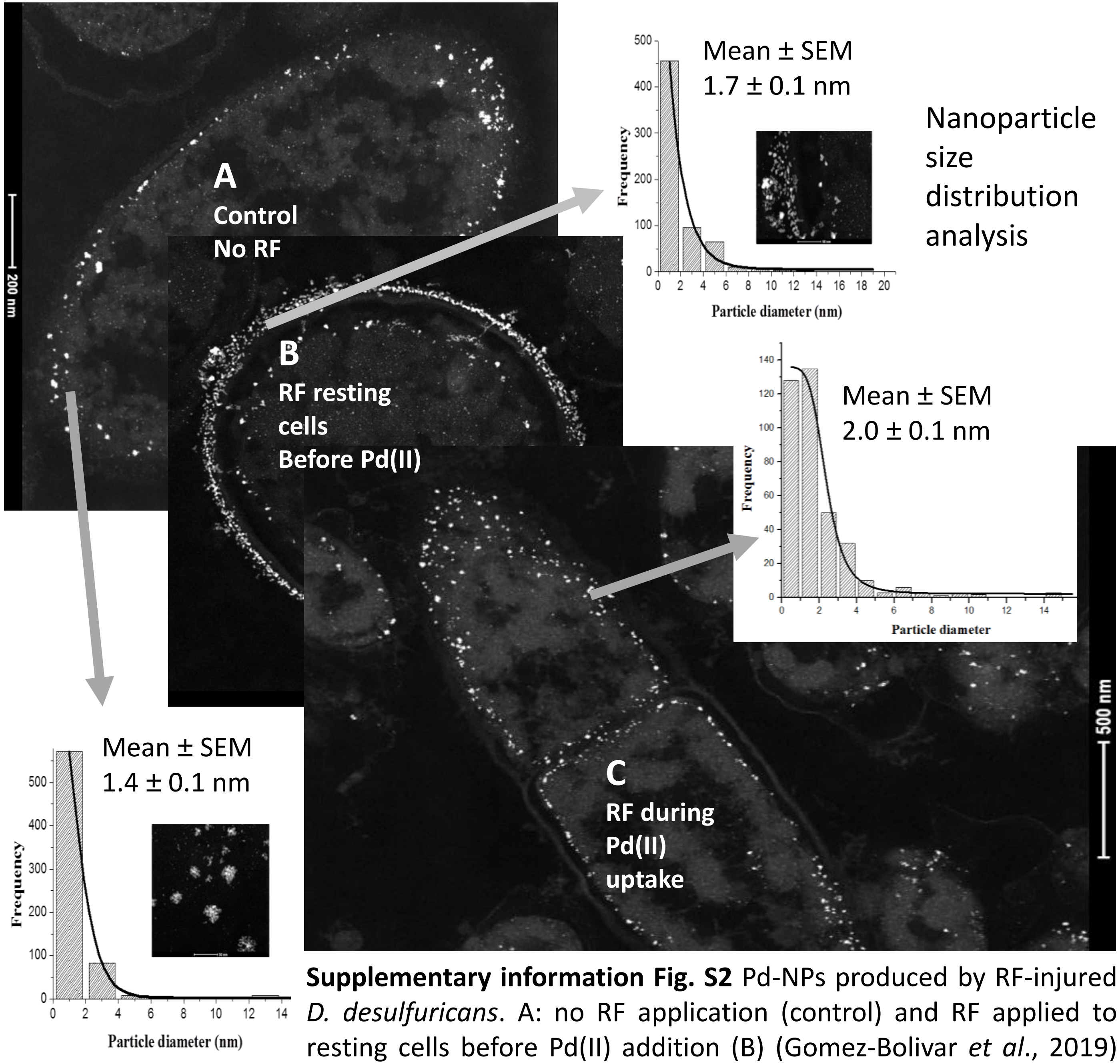

Supplementary information Fig. S2 Pd-NPs produced by RF-injured *D. desulfuricans*. A: no RF application (control) and RF applied to resting cells before Pd(II) addition (B) (Gomez-Bolivar *et al.*, 2019) or during Pd(II) uptake (C). Dispersivity indexes were A: 2.07; B: 1.26; C: 0.98 (calculated according to Gomez-Bolivar *et al.*, 2019).
