# Supplementary material for: Enhanced hydrogenation catalyst synthesized by Desulfovibrio desulfuricans exposed to a radio frequency magnetic field
Source: Microb Biotechnol. 2021 Jul 3;14(5):2041–58. doi: 10.1111/1751-7915.13878 (PMC8449679; doi:10.1111/1751-7915.13878)
Supplement: Supplementary file 3 — Fig. S3. Effect of RF treatment on Pd‐NP formation. [file MBT2-14-2041-s004.pdf]

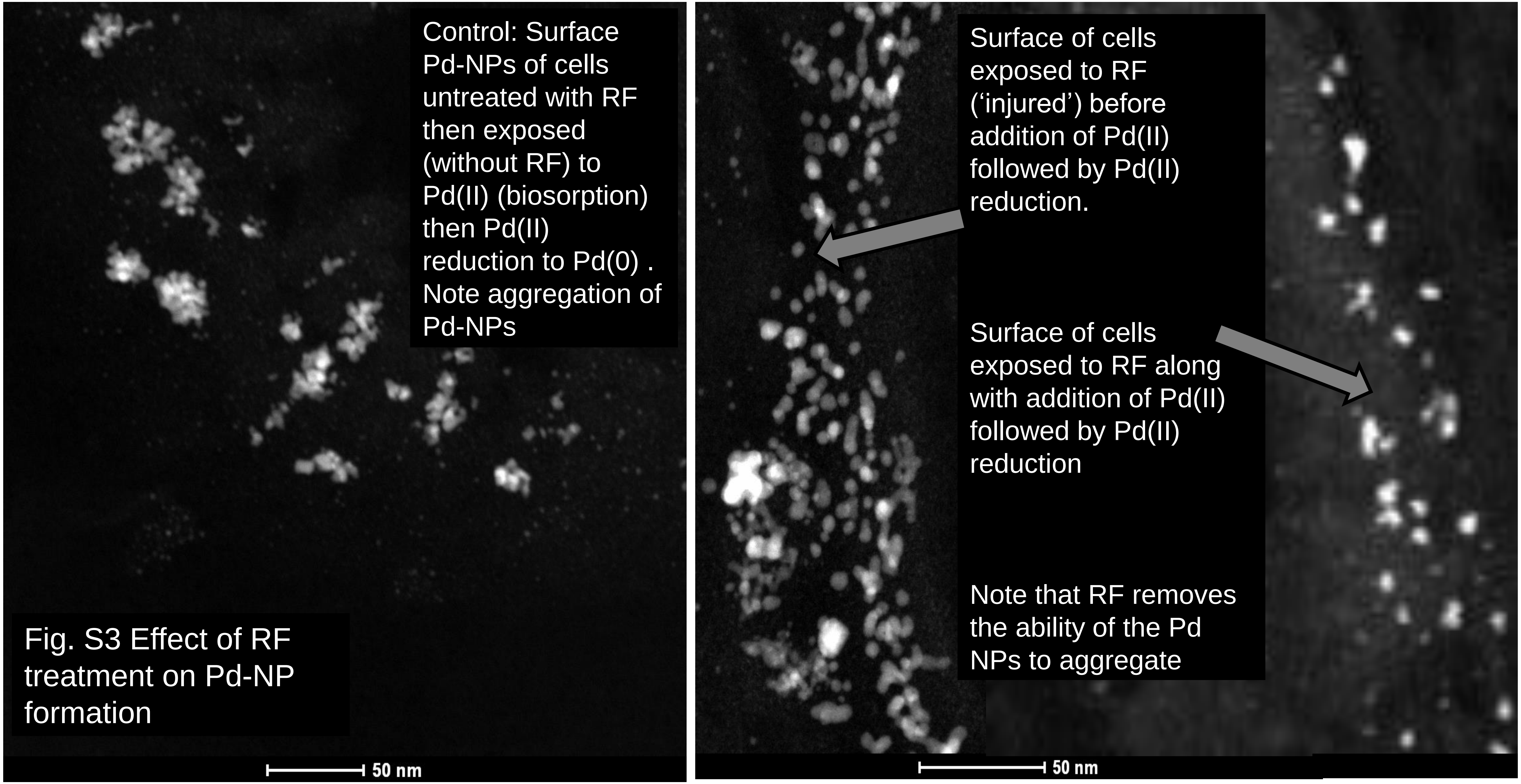

Fig. S3 Effect of RF treatment on Pd-NP formation
