# Supplementary material for: Enhanced hydrogenation catalyst synthesized by Desulfovibrio desulfuricans exposed to a radio frequency magnetic field
Source: Microb Biotechnol. 2021 Jul 3;14(5):2041–58. doi: 10.1111/1751-7915.13878 (PMC8449679; doi:10.1111/1751-7915.13878)

Supplementary Information 4. Magnetic imaging of cells loaded to 5wt% Pd(0)

Examination of Pd-loaded cell population by X-ray scanning microscopy

This recently-developed method uses X-rays emitted from the sample under a synchtrotron radiation beam. By use of a polarized beam magnetic information can be obtained by interactions with the magnetic field emitted by the sample. Preliminary tests to visualize the population by location of palladium by its X-ray emission showed individual cells to be below the limit of resolution, both by the sensitivity of detection and by pixellation (see Figure). As the cells are approximately 2 microns in length (see below) they are not clearly discernable by this method. Hence, mapping and enumeration of the population using magnetic imaging was not attempted; the method is still under development at Diamond Light Source.

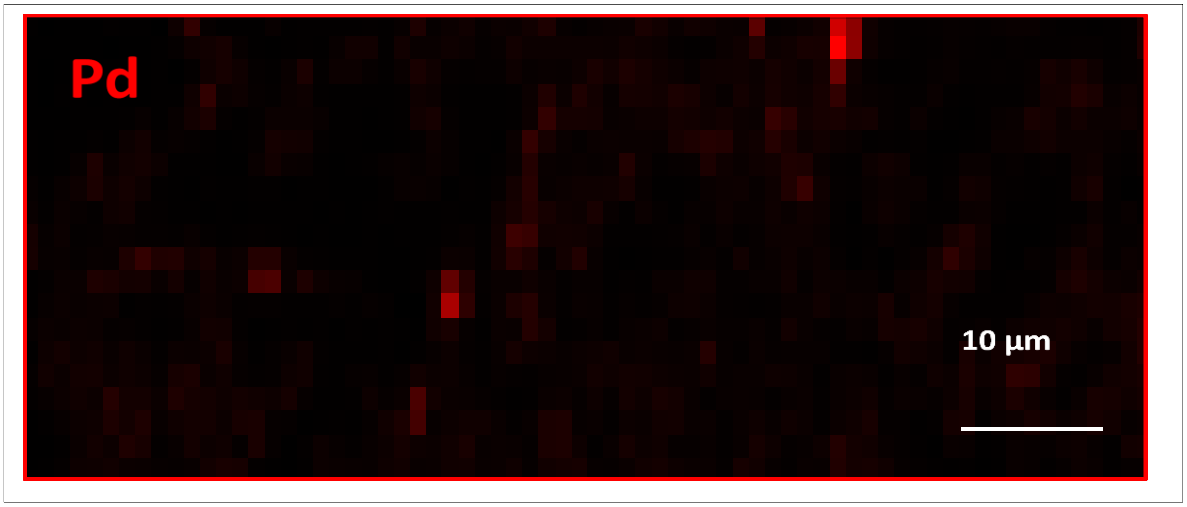

Examination of single cells by magnetic force microscopy

Palladized cells of *D. desulfuricans* were examined initially using atomic force microscopy as described by Omajali (2015) to obtain images as shown in native cells (Figure 2 top, left) and cells loaded to 5wt% Pd (Figure 2 top, right). Note that the height is variable, attributable to partial collapse of the heavily hydrated extracellular polymeric material, upon examination of native cells in air. The resolution is insufficient to visualize individual Pd-NPs (of sizes of a few nm) on individual cells. Scale shown on the X and Y axes is microns. Height is as shown inset.

By using a magnetic tip an image is obtained of the magnetic domains on a single cell. Note that the method does not visualize individual Pd-NPs but the perturbations they induce in the magnetic field ~20nm above the sample (as probed by the tip rastered across the sample at this distance). The diagram (Figure, bottom left) shows how the Pd(0) nanocrystals are seen indirectly, as lines of magnetic force, imaged in false colour on the cells (Figure, bottom right). As with X-ray scanning microscopy (above), the resolution is insufficient to obtain useful information by this method.

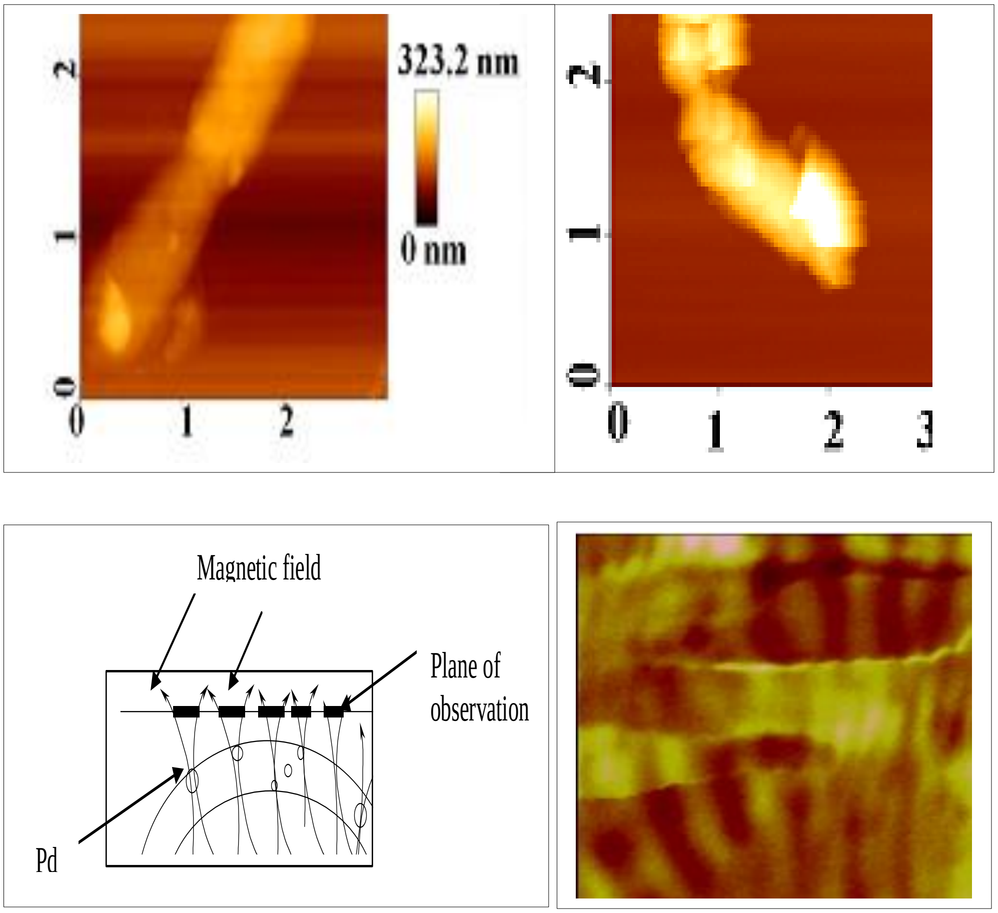

Supplement: Supplementary file 4 — Fig. S4. Magnetic imaging of cells loaded to 5wt% Pd(0). [file MBT2-14-2041-s003.pdf]
