# Supplementary material for: Enhanced hydrogenation catalyst synthesized by Desulfovibrio desulfuricans exposed to a radio frequency magnetic field
Source: Microb Biotechnol. 2021 Jul 3;14(5):2041–58. doi: 10.1111/1751-7915.13878 (PMC8449679; doi:10.1111/1751-7915.13878)
Supplement: Supplementary file 5 — Fig. S5. Mobile phone ‘communication’ with E. coli perturbs the expression of genes involved in fundamental cellular processes. [file MBT2-14-2041-s005.pdf]

Supplementary information S5. Mobile phone 'communication' with *E. coli* perturbs the expression of genes involved in fundamental cellular processes

Steven J. Megit, Colin Berry and Andrew P. Morby (unpublished work, shown with permission)
Cardiff School of Biosciences, Cardiff University Museum Avenue, Cardiff CF10 3US, UK

Fold Change in Transcript Abundance	Gene Designation	Gene Product Function
-4.6	*yacG*	Unknown
+4.0	*minE*	Cell division factor *Reverses MinC inhibition of ftsZ ring formation*
+3.9	*holA*	DNA-replication/repair, restriction/modification
+3.2	*insA_1*	Transposon-related functions
+4.6	*nohA*	Lambda terminase homologue
+2.4	*ydgR*	Putative transport protein
+2.2	*yohG*	Putative channel / filament protein
+2.4	*yifK*	Putative amino acid / amine transport protein
+3.0	*nrfE*	Energy metabolism *Formate-dependent nitrite reductase; possible assembly function*
**+3.1**	***hyaF***	**Energy metabolism *Nickel incorporation into hydrogenase-1 proteins***
**+3.2**	***hyfI***	**Energy metabolism *Hydrogenase 4Fe-S subunit***
**+3.6**	***nikD***	**Transport of small molecules *ATP-binding protein of nickel transport system***
+1.8	*betT*	High-affinity choline transport

Table 1 Genes with altered transcript abundance in response to mobile phone radiation
Only those genes are listed for which the alteration (increase (+) or decrease (-)) in transcript abundance was at least two standard deviations greater than the mean value calculated in each case from three independent experiments. The value presented is the average fold-alteration in comparison with control cells. Data show that 13 genes undergo significant alterations in expression on exposure to radiation from a mobile phone. The changes in transcript abundance were detected for genes involved in cell division, DNA replication, phage mobilisation and transposition, so that mobile phone radiation appears to perturb fundamental cell processes, including three functions involved in nickel transport and metabolism (bold: see main text for relevance).

Methods: *E. coli* strain TG1 was grown in LB medium (100 ml) to an A_{600} of 0.040 ± 0.005. The culture was divided (30ml $flask^{-1}$) between two 500 ml conical flasks, to one of which, a 900MHz GSM digital mobile phone (MAXON 3204: 2W maximal output) was attached externally (inverted to increase the proximity of the aerial to the growing culture). The phone was activated to transmit and both cultures were grown in separate but identical incubators with shaking (200 rpm) for two hours at 37°C. Cultures were confirmed to be isothermal using a Hanna Instrument HI93510 thermometer and liquid probes. Cells were harvested (A_{600} 0.46 ± 0.01) and total RNA was extracted immediately. Transcript abundance was determined using duplicate Sigma-Genosys Panorama *E. coli* gene arrays and quantified with a BIO-RAD Molecular Imager FX phosphoimager/Quantity One software. The experiments were performed in triplicate and to control for possible variations between incubators and/or the individual filters in a duplicate pair, these were alternated between non-irradiated control and irradiated cultures in sequential experiments.

Details and wider relevance: Ubiquitous mobile phone usage world-wide has led to concerns about possible health effects from the use of a microwave emitter in proximity to live cells.

Microwave energy was shown to induce alterations in cellular function both in whole organisms and in isolated neural tissues[1,2], but such studies often employ defined microwave sources (transverse electromagnetic cells) and exaggerated power levels. We exposed cells of a model system (*E. coli* TG1) to radiation emitted by a commercially-available mobile phone. By employing array technology, the

transcriptional levels of all identified open reading frames in the genomic sequence of this organism[3] were measured.

The transcript abundance of 13 from 4255 genes (Table 1) was significantly altered (>95% confidence limit) on exposure to mobile phone radiation in each of 3 separate experiments. One gene (*yacG*) which encodes a product of unknown function exhibited diminished expression, whereas increased transcript levels were detected for 12 genes, 8 of which encode known or predicted membrane associated proteins. These can be functionally classified into cell proliferation (MinE, HolA), DNA mobilisation (InsA, NohA), transport (NikD, BetT), putative transport (YdgR, YohG, YifK) and energy metabolism (NrfE, HyaF, HyfI).

The protein product of the cell cycle-associated gene *minE* stimulates cell-division at internal division sites whilst suppressing secondary division sites which form at the poles of an *E. coli* cell, thereby generating topological specificity. The *holA* gene encodes the delta sub-unit of DNA polymerase III and since this enzyme is the major replicative complex in *E. coli*, the increased transcript abundance for *minE* and *holA* may both reflect alterations in cell division. These findings are in keeping with those previously reported[4,5] which suggested an increase in the division rate of eukaryotic cells exposed to microwave energy.

Many copies of insertion elements (IS) are present within *E. coli* DNA and their movement is capable of driving rearrangement by recombination within the genome. Increased expression of *insA* was detected in response to mobile phone radiation and, again, this is consistent with elevated transcription of IS genes in *E. coli* cells in response to stress[6]. Another enzyme involved in DNA scission is NohA which cleaves replicating phage genomes into linear fragments with prior to association with phage coat proteins. Both InsA and NohA are capable of generating “free” DNA termini *in vivo*. Chromosomal damage has been shown to occur in eukaryotic cells exposed to microwaves but it was postulated that this damage did not occur by direct absorption of the microwave energy by DNA[7,8]. The induction of genes which represent functional analogues of *insA* and/or *nohA* in mammalian systems may explain the chromosomal damage observed due to microwave exposure.

Three genes encoding hydrogenase associated proteins (NrfE, HyaF, HyfI) were induced in this study together with *nikD* encoding a Ni(II)-export ATPase. These 4 genes are functionally associated given the Ni(II) requirement of many membrane-associated hydrogenases[9]. Osmotic stress and a range of other conditions e.g. growth under anaerobiosis, has been shown to modulate the expression of hydrogenase genes in *E. coli*[10,11]. An increase in medium osmolarity has been shown previously to result in elevated transcription of the *betT* gene which encodes a choline import protein[12] (glycine-betaine, which acts as a neutral osmolyte in these cells, is synthesised from the precursor, choline). An increase in *betT* expression was seen in response to mobile phone radiation. Thus, induction of *nrfE*, *hyaF*, *hyfI, nikD* and *betT* by mobile phone radiation may be linked to alterations in membrane status leading to changes in the perception of osmotic potential or actual changes in cytoplasmic ion composition. Indeed, in mammalian cells, membrane integrity and/or membrane transport, including choline import in neural tissue[13], has been reported to be affected by exposure to microwave radiation[14,15].

These experiments provide incontrovertible proof of significant change in gene expression in response to mobile phone radiation. In addition the changes observed have direct parallels with reported effects on eukaryotic cells elicited by similar exposure.

References **1.** Preece, A.W., *et al., Int. J. Radiation Biology* **75**, 447-456 (1999). **2.** Tattersall, J.E.H., Wood, S.J. & Scott, I.R. *Proc IEE Seminar, Electromagnetic assessment and antenna design relating to health implications of mobile phones London* 5/1-5/4 (1999). **3.** Blattner, F.R., *et al., Science* **277**, 1453-1474. (1997). **4.** Daniells C., *et al.Mutat. Res.* **399,** 55-64. (1998). **5.** Velizarov, S. Raskmark, P. & Kwee, S. *Bioelectrochem. Bioenerg.* **48,** 177-180 (1999). **6.** Naas, T., Blot, M., Fitch, W. M. & Arber, W. *Genetics* **136**, 721-730 (1994). **7.** Lai, H. & Singh, N.P. *Bioelectromagnetics* **18**, 446-54 (1997). **8.** Saffer, J.D. & Profenno, L.A. Bioelectromagnetics. **13**, 75-78. (1992). **9.** Wu, L.F. & Mandrand, M.A. *FEMS Microbiol. Rev.* **10**, 243-69 (1993). **10.** NiBhriain, N., Dorman, C.J. & Higgins, C.F. *Molec. Microbiol.* **3**, 933-944 (1989). **11.** Gousebet, G., *et al J. Bacteriol.* **175**, 214-221 (1993). **12.** Lamark T., *et al. Molec. Microbiol*. **5,** 1049-1064 (1991). **13.** Lai, H., *et al. Pharmacol. Biochem. Behav.* **33**, 131-138 (1989). **14.** Dwivedi, R.S. Dwivedi, U. & Chiang, B. *Exp. Cell Res.* **180**, 253-265 (1989). **15.** Geletyuk, V.I., *et al. FEBS Lett.* **359**, 85-88 (1995).
